# The BH3-only protein Bad is dispensable for TNF-mediated cell death

**DOI:** 10.1038/cddis.2014.575

**Published:** 2015-01-22

**Authors:** E Ottina, M Sochalska, R Sgonc, A Villunger

**Affiliations:** 1 Division of Developmental Immunology, Biocenter, Medical University Innsbruck, Innsbruck, Austria; 2 Division of Experimental Pathophysiology and Immunology, Biocenter, Medical University Innsbruck, Innsbruck, Austria

## Abstract

Tumor necrosis factor (TNF) is a key signaling molecule orchestrating immune and inflammatory responses and possesses the capacity to trigger apoptotic as well as necroptotic cell death. Apoptotic cell death elicited by TNF has been demonstrated to engage pro-apoptotic Bcl-2 family proteins, most prominently the BH3-only protein Bid, a key substrate of caspase-8, the key effector protease downstream of TNF receptor I. Most recently, the BH3 domain-containing protein Bad (Bcl-2-antagonist of cell death) has been shown to be rate limiting for TNF-mediated cell death, suggesting possible synergy with Bid, but genetic analyses presented here demonstrate that it is dispensable for this process.

Tumor necrosis factor (TNF) is a pleiotropic cytokine critical for inflammation during innate and adaptive immune responses, orchestrating cytokine expression via activation of the canonical NF-*κ*B (nuclear factor kappa-light-chain-enhancer of activated B cells) signaling cascade. TNF can also trigger cell death by engaging TNF receptor I (TNFRI) along apoptotic or necroptotic pathways, which is believed to limit or exacerbate inflammatory responses, respectively.^1^ Excessive TNF release during fulminant bacterial infection can lead to a cytokine storm, vascular leakage driven by TNF-mediated endothelial damage, septic shock and death.^2^ The pro-death and inflammatory potential of TNF therefore need to be tightly regulated and cells have developed highly specialized means to control the biological outcome of TNFRI activation.

Recent studies define a delicate network of linear and branched ubiquitylation/deubiquitylation-controlled signaling events downstream of TNFRI as critical for the decision between life and death upon TNF stimulation.^3, 4^ Its pro-death function is thereby constrained by different means but most prominently by NF-*κ*B-dependent transcription of survival genes, such as Bcl-x, XIAP (X-linked inhibitor of apoptosis protein), cIAPs (inhibitor of apoptosis proteins) or c-FLIP (cellular FLICE inhibitory protein), a dimerization and activation partner of procaspase-8 that is rate limiting for TNF-driven apoptosis and also prevents RIPK1/RIPK3-dependent necroptosis that can ensue when caspase-8 is inhibited.^5^

To promote death, TNFRI signaling can also engage pro-apoptotic members of the Bcl-2 family, for example, the BH3-only proteins Bim, Bid, Puma and Bad (Bcl-2-antagonist of cell death). Bid is processed and thereby activated by caspase-8 in many cell types and systems. Bim activity, on the other hand, appears to be controlled by TNF-driven JNK-mediated phosphorylation and both, Bid and Bim, contribute to T-cell-driven TNF-dependent liver toxicity.^6^ TNF has also been shown to trigger NF-*κ*B-dependent transcriptional activation of Puma and deficiency of this BH3-only protein can also provide protection from hepatocyte killing upon systemic TNF administration.^7^ Together these findings show that mitochondrial apoptosis, controlled by the Bcl-2 family, can be a critical event in pathological hepatocyte cell death in response to TNF.

Bad phosphorylation on residues Ser-112, -136, -155, has previously been shown to prevent mitochondrial translocation of Bad and restrain its pro-death function (reviewed in Danial^8^). More recently, Yan *et al.*^9^ showed that the activity of the BH3 domain-containing protein Bad is repressed downstream of TNFRI activation by direct IKK-mediated phosphorylation on Ser-26. This study showed that mouse embryonic fibroblasts (MEFs), derived from *Ikbkb*^*−/−*^ mice, that are deficient in IKK*β*/IKK2, where Bad expression was also repressed by RNA interference, or MEFs from *Bad*^*−/−*^ mice that were pre-treated with the IKK inhibitor PS-1145, showed reduced caspase-3/7 activity, delayed PARP cleavage and strongly reduced cell death upon TNF treatment. Similar observations were made in primary thymocytes and hepatocytes, pretreated with IKK-inhibitor prior to TNF stimulation. Most strikingly, Bad-deficient animals were protected from the lethal effects of hepatitis elicited by D-GalN priming and subsequent TNF injection. Together, these data provided a strong evidence for an NF-*κ*B-independent anti-apoptotic function of the IKK complex, explaining, at least in part, the increased cell death susceptibility of IKK*β*-deficient MEF over those lacking the NF-*κ*B family members RelA plus cRel^9^ and supported a rate-limiting role of Bad in TNF-induced cell death signaling.

Intrigued by an earlier report describing the generation and phenotype of *Bad*^*−/−*^ mice that reported normal cell death responses upon TNF treatment,^10^ we decided to reinvestigate the role of Bad in TNF-driven cell death *ex vivo* and in fulminant hepatitis.

## Results

### The BH3-only protein Bad is dispensable for TNF killing upon IKK inhibition

**Figure 1:**
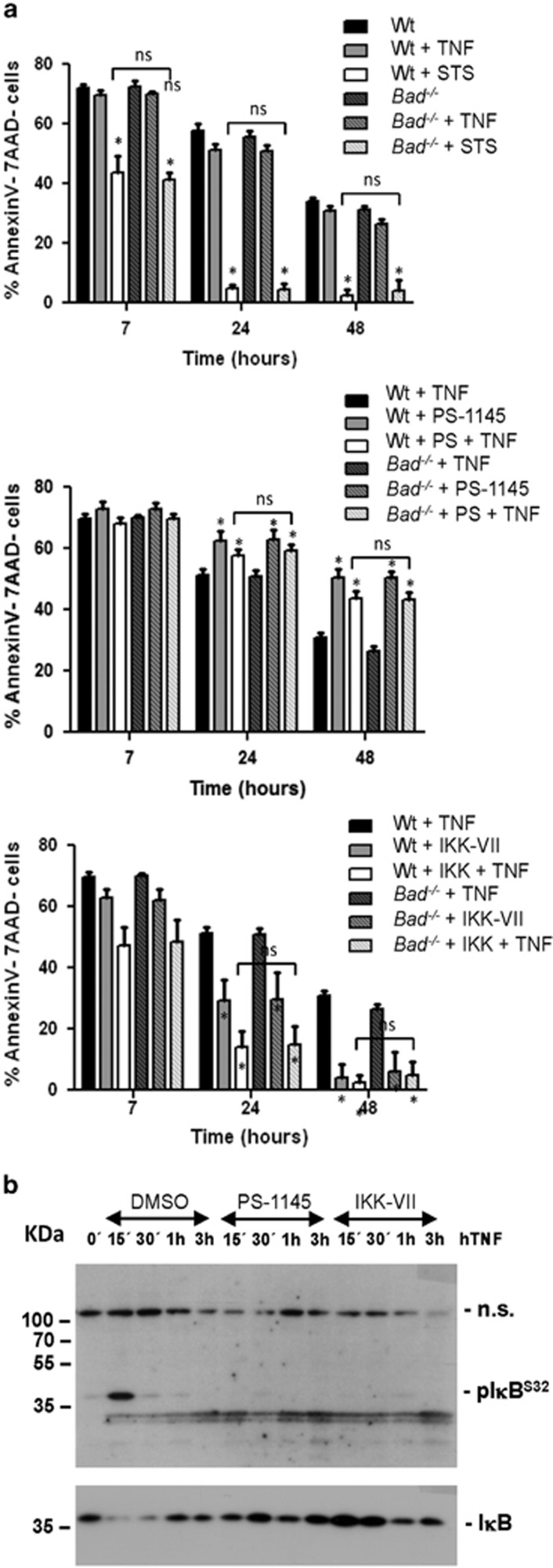
PS-1145-mediated inhibition of IKK fails to sensitize thymocytes to TNF killing. (**a**) Thymocytes from wt or *Bad*^*−/−*^ mice 6-12 weeks of age were put in culture, left untreated or were pretreated with the IKK inhibitor PS-1145 (10 *μ*M) or IKK-VII (10 *μ*M) for 2 h prior TNF (5 ng/ml) simulation. Staurosporine (STS) was used at 100 nM as a control. Viability was assessed over time by Annexin V plus 7-AAD staining and flow cytometry. Bars represent means±S.E.M. of *n*=6 independent experiments performed in triplicates. (**b**) Thymocytes from *Bad*^*−/−*^ mice were treated with solvent or IKK inhibitors (10 *μ*M) for 2 h prior TNF stimulation (5 ng/ml) and analyzed by western blot to confirm the activity of IKK inhibitors

First, we isolated thymocytes from wt or *Bad*^*−/−*^ animals and exposed them to the IKK inhibitor PS-1145 or an alternative inhibitor, IKK-VII, followed by the administration of TNF. For control purposes, staurosporine (STS) was used as an unrelated cell death inducer. Cell death was assessed by Annexin V and 7-AAD staining and flow cytometric analysis. In contrast to published findings,^9^ we were unable to detect a survival difference between wt and *Bad*^*−/−*^ thymocytes. In fact, we did not see a sensitization of thymocytes toward TNF killing by PS-1145 pretreatment. If anything, this inhibitor delayed spontaneous cell death of thymocytes in culture at later time points, while IKK-VII treatment sensitized thymocytes to spontaneous cell death in culture as well as TNF killing. However, wt and *Bad*^*−/−*^ thymocytes responded at similar rates (Figure 1a,Supplementary Figure 1). Both inhibitors effectively prevented NF-kB activation, as monitored by western analysis, using antibodies for pIkB^S32^ or total IkB, that becomes phosphorylated and degraded upon TNF treatment (Figure 1b). We conclude that Bad does not contribute to spontaneous or TNF-mediated cell death in thymocytes, neither under steady state conditions, in line with the initial reports by Ranger *et al.*,^10^ nor upon inhibition of the IKK complex (this study).

**Figure 2:**
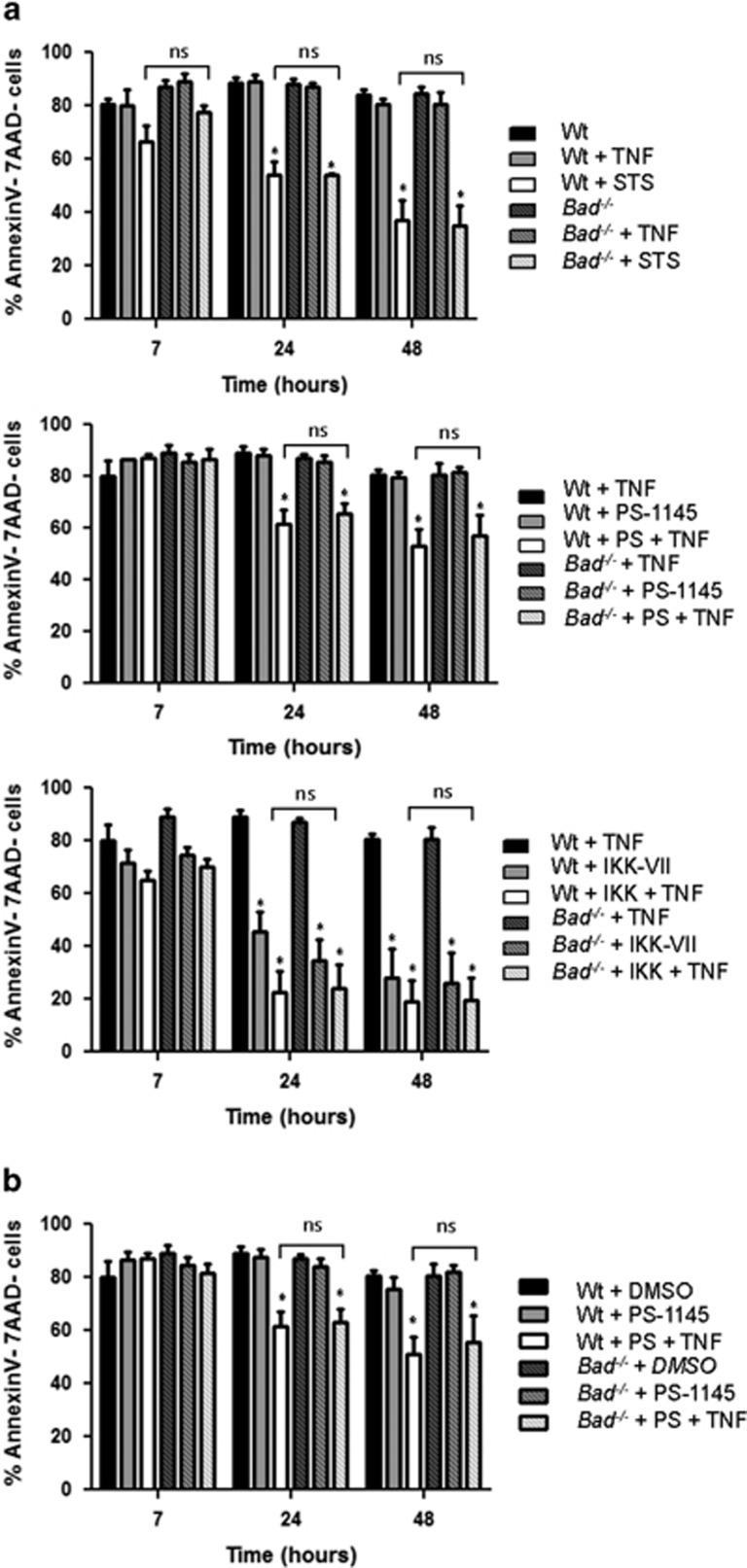
Loss of Bad does not protect from TNF killing after IKK inhibition in SV40 MEF. (**a**) SV40 immortalized MEF were treated and analyzed as described in Figure 1. Bars represent means±S.E.M. of *n*=6 independent experiments using six individual batches of E13.5-derived embryos per genotype, subsequently immortalized with SV40 LT. (**b**) Dose escalation of PS-1145 (25 *μ*M) does not further enhance TNF (5 ng/ml) killing in SV40 MEF. Bars represent means±S.E. of *n*=6 independent experiments using six individual batches of SV40 MEF per genotype

Next, we investigated cell death responses in a series of independent batches of SV40 large T antigen-immortalized MEFs, because in our experience even wild-type MEFs can be highly variable in their response to different cell death triggers. However, all batches of SV40 MEFs, whether from wt or *Bad*^*−/−*^ cells, were equally sensitive to STS or IKK inhibition in the absence or presence of TNF. In contrast to the situation in thymocytes, PS-1145 sensitized MEF to TNF, in line with a prosurvival effect of NF-*κ*B-regulated genes, and/or, modification of alternative direct targets by IKK in these cells. However, absence of Bad failed to protect from or delay cell death under conditions of IKK inhibition (Figure 2a, Supplementary Figure 1). To exclude issues of batch dependence or dose dependence of the inhibitor used, we also did a dose escalation study raising the inhibitor concentration from 10 to 25 *μ*M (Figure 2b) and tested PS-1145 from a different provider (Supplementary Figure 2A), confirming our initial results. As the mode of immortalization may affect responsiveness to TNF, we also investigated the response of primary low passage MEF derived from E14.5 embryos. Also, in these cells, PS-1145 treatment failed to sensitize the cells to TNF killing, while the alternative inhibitor IKK-VII was highly toxic at the concentration used (Supplementary Figure 2B).

### Loss of Bad does not ameliorate fulminant hepatitis by GalN/TNF treatment

**Figure 3:**
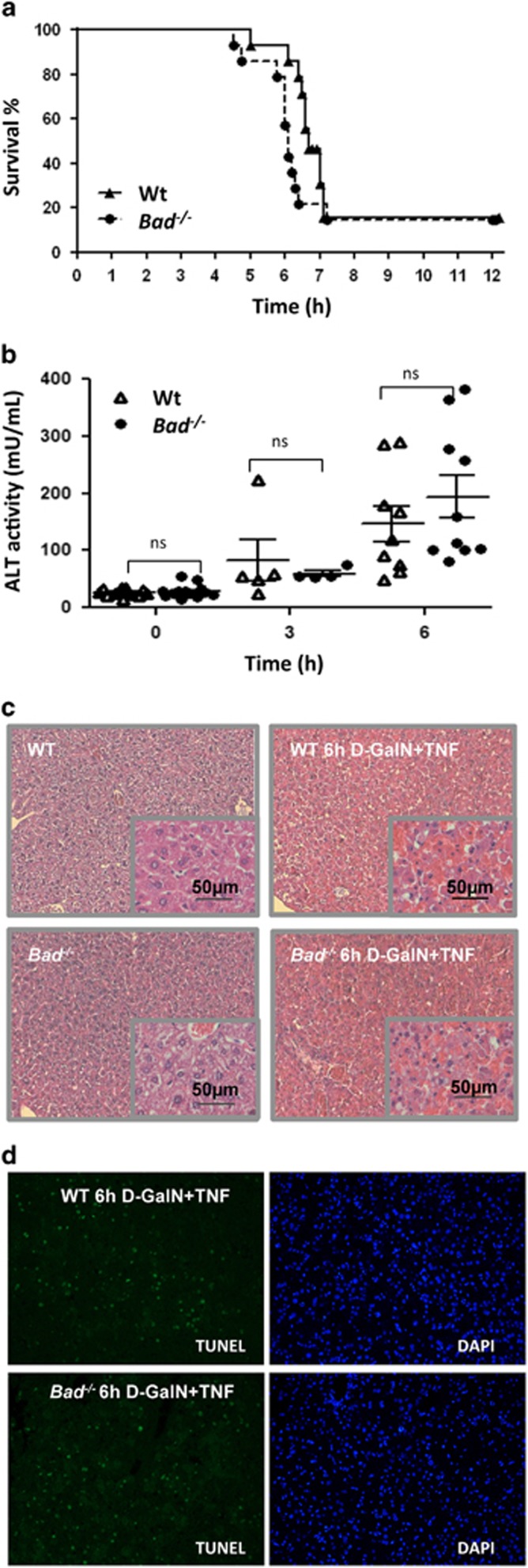
Loss of the BH3-only protein Bad does not protect from fulminant hepatitis. (**a**) Mice of the indicated genotypes were sensitized with D-GalN prior TNF treatment for hepatitis induction (*n*=10 from two independent experiments using five mice per genotype). (**b**) Sera were analyzed for ALT content by ELISA and organs were fixed in 4% PFA and processed for (**c**) H&E or (**d**) TUNEL staining

Puzzled by these findings, we decided to trigger fulminant hepatitis by GalN priming and subsequent TNF injection, although it remained unclear to us how inhibition of RNA synthesis by GalN-mediated UTP depletion, globally compromising protein synthesis in hepatocytes^11^ should mimic the selective inhibition of IKK kinase activity. Consistent with our *in vitro* analyses, wild-type and Bad-deficient mice succumbed to fulminant hepatitis in a comparable manner, whether assayed by mean time to death, release of alanine aminotransferase (ALT) liver enzyme, histological assessment or TUNEL staining of liver sections of these mice (Figure 3).

## Discussion

Using different primary and immortalized cells lacking the BH3-only protein Bad, as well as an *in vivo* model of fulminant hepatitis, we failed to detect a significant contribution of this pro-apoptotic protein to TNF killing. Our observations argue strongly against a rate-limiting role of Bad in TNF-mediated cell death, contrasting a previous report.^9^ A possible explanation for our divergent results with thymocytes (Figure 1,Supplementary Figure 1) may be attributed to differences in genetic background of the animals used (C57BL/6N in our study *versus* an undefined background). A comparison between C57BL/6N and C57BL/6J mice did not reveal significant differences in survival upon GalN/TNF treatment (not shown). Genetic background differences, however, have been shown to impact on the activity of cell death related genes (reviewed in Manzi *et al.*^12^). Differences in MEF sensitivity might be explained, next to genetic background issues, by different modes of immortalization. Although we used six independently generated batches of SV40 large T antigen-immortalized MEF per genotype (Figure 2), generated fresh in our lab, Yan *et al.* do not specify how their fibroblasts were immortalized or whether they used primary MEF, as used in the original paper describing *Bad*^*−/−*^ mice.^10^ Hence, we also tested three independent batches of primary low-passage E14.5 MEF but found no difference between wild-type and *Bad*^*−/−*^ cells (Supplementary Figure 2), although, admittedly, these cells were poorly sensitized to cell death by IKK inhibition. Genetic background may also account for differences in the *in vivo* responsiveness of Bad-deficient animals to TNF-induced hepatitis (Figure 3), as backcrossing in different facilities will ultimately lead to the retention of different polymorphisms form the SV129 genetic background on which these mice were originally made. Gender may also affect the inflammatory response during sepsis with females being more vulnerable.^13, 14^ We therefore balanced the number of male and female mice in our study. Neither age nor sex were specified in the publication by Yan *et al.*,^9^ while we avoided such a possible bias. Finally, we also noted that, although ALT release is a reliable marker for liver damage, TUNEL analysis can yield quite variable results depending on the section plane of the tissue analyzed, that shows frequently patchy distribution of TUNEL^+^ cells coinciding with necrotic lesions in hematoxylin and eosin staining, rather than homogenous distribution across the whole field (Supplementary Figure 3).

Our results do not address whether Bad is an IKK substrate but rather question its physiological relevance in TNF killing. A protective phosphorylation of the BH3-only protein PUMA by the IKK complex, downstream of the IL-3 receptor was recently reported,^15^ but mutation of Ser-10, despite increasing PUMA half-life experimentally, had only minor impact on cell viability.^16^ Along similar lines, phospho-defective knock-in mouse mutants of Bim, lacking serine residues described to decrease protein stability and apoptotic potential upon phosphorylation, show no overt phenotype and normal Bim-dependent cell death responses, suggesting that the relative increase of Bim noted under these conditions is not of significance in the systems where it has been tested.^17^ Similarly, JNK target-sites in the BH3-only proteins Bim and Bmf, proposed to promote their apoptotic potential, were shown to be largely dispensable for cell death signaling *in vivo*.^18, 19^ Together, these studies suggest that the phosphorylation of BH3-only proteins by kinases such as AKT, ERK, JNK or IKK may represent initial priming events that rely on additional signaling input in order to convert posttranslational modifications into quantifiable biological outputs of physiological relevance. However, sometimes they may simply represent bystander events of negligible significance. *In vitro* experiments are often designed to highlight small differences and may possibly reveal quite sophisticated regulation of signaling that, in the context of an *in vivo* response, is overwhelmed. Thus it remains possible that Bad phosphorylation has a role in regulating cell death outcomes but our evidence suggests that in widely accepted TNF signaling paradigms it does not have a significant role.

## Materials and Methods

### Mouse work

The generation of *Bad*^*−/−*^ *mice* has been described.^10^ Animals were maintained in C57BL/6N genetic background after backcrossing and compared with age and gender-matched wild-type C57BL/6N and C57BL/6J mice. Animal experiments were performed in accordance with Austrian legislation (BMWF-66.011/0068-II/3b/2014). Genotype-blinded animals, 8–12 weeks of age, were sensitized by administration of 700 mg/kg body weight of D-GalN (Sigma, Vienna, Austria) i.p. and 30 min later treated with 15 mg/kg body weight of hTNF (Peprotech, London, UK) along the same route. Mice were monitored up to 12 h post TNF challenge, liver specimens were collected at the time of death and fixed for the preparation of histological sections. Blood was collected from the submandibular vein.

### Cell culture and reagents

Thymocytes or MEF, derived from E13.5 embryos, were cultured in DMEM (PAA Laboratories, Linz, Austria), 250 *μ*M L-glutamine (Gibco, Vienna, Austria), 50 *μ*M 2-mercaptoethanol, penicillin/streptomycin (Sigma) and 10% fetal calf serum (PAA Laboratories). Staurosporine (Sigma) was used at 100 nM, IKK inhibitors IKK-VII and PS-1145 were purchased from Sigma or Calbiochem (Vienna, Austria), for reference purposes. Cells were pretreated with 10 or 25 *μ*M of inhibitors for 2 h prior addition of 5 ng/ml or 25 ng/ml hTNF (Peprotech). Antibodies for western analysis were purchased from Cell Signaling (Beverly, MA, USA).

### Histology

Liver lobes were fixed in 4% paraformaldehyde in PBS. Tissues were processed according to standard procedures, cut in 3 μm sections and stained in hematoxylin and eosin or were processed for TUNEL staining by immunofluorescence.

### TUNEL staining

Liver tissue embedded in paraffin was cut at 3 *μ*m, deparaffinized and rehydrated. For visualization of apoptotic cells via the *in situ* terminal transferase-mediated dUTP nick end-labeling method (TUNEL, Roche, Manheim, Germany), rehydrated slides of tissue sections on cover slips were treated with 0.1% Triton X-100/0.1% sodium citrate buffer and dehydrated by graded series of alcohol and chloroform. The TUNEL reaction was carried out in a humidified chamber for 1 h at 37°C using recombinant terminal transferase and FITC-dUTP (Roche) as described.^20^ Counterstaining of nuclei was done with DAPI (Sigma).

### Statistics

Two-way ANOVA with Bonferroni post-test was used for statistical analysis. A *P*-value <0.05 was considered to be statistically significant.

## Abbreviations

TNF: tumor necrosis factor
NF-*κ*B: nuclear factor kappa-light-chain-enhancer of activated B cells
Bad: Bcl-2-antagonist of cell death
XIAP: X-linked inhibitor of apoptosis protein
IAP: inhibitor of apoptosis protein
c-FLIP: cellular FLICE inhibitory protein
