# Correction to: The BH3-only protein Bad is dispensable for TNF-mediated cell death

**DOI:** 10.1038/s41419-018-0947-5

**Published:** 2018-09-05

**Authors:** E. Ottina, M. Sochalska, R. Sgonc, A. Villunger

**Affiliations:** 1 Division of Developmental Immunology, Biocenter, Medical University Innsbruck, Innsbruck, Austria; 2 Division of Experimental Pathophysiology and Immunology, Biocenter, Medical University Innsbruck, Innsbruck, Austria

*Correction to*: *Cell Death and Disease* **6:**e1611; 10.1038/cddis.2014.575; published online 22 Jan 2015

Since publication of this article, the authors wished to draw attention to an error in the materials section as a result of which they have been mis-cited (https://www.nature.com/articles/s41422-018-0041-7). The dose of TNF given was not in fact 15 mg/kg body weight (as stated in the “mouse work” section), but 15 μg/kg body weight.

The authors would like to apologize for any inconvenience this may have caused.

