# Supplementary material for: The BH3-only protein Bad is dispensable for TNF-mediated cell death
Source: Cell Death Dis. 2015 Jan 22;6(1):e1611–. doi: 10.1038/cddis.2014.575 (PMC4669773; doi:10.1038/cddis.2014.575)
Supplement: Supplementary Figures [file cddis2014575x1.pdf]

The BH3-only protein Bad is dispensable for TNF-mediated cell death

- **Supplemental Figure legends:**

- **Figure S1:** Representative dot plots of wt and *Bad*$^{-/-}$ thymocytes or SV40 immortalized MEF treated as described in Figure 1. Cells were stained with 7-AAD and Annexin V-FITC after 24h in culture and analyzed by flow cytometry.

- **Figure S2:** (A) PS-1145 from different commercial providers (PS-a vs. PS-b) yield similar sensitization of SV40 MEF to TNF killing using different concentration of inhibitor (2h pre-treatment) and graded doses of TNF. Bars represent means ± SD of n=2 independent experiments using three individual batches of SV40-MEF per genotype in each experiment. (D) Primary low passage E13.5 MEF (p<5) were pre-treated with PS-1145 or IKK-VII ± TNF. Bars represent means of a three experiments performed using MEF from three individual embryos. Viability was assessed over time using Annexin V plus 7-AAD staining.

- **Figure S3:** TUNEL vs. DAPI staining of liver section of individual wt and *Bad*$^{-/-}$ mice moribund after D-GalN and TNF treatment reveals significant heterogeneity in individual specimens, but no evidence for consistent differences between genotypes. (T) indicates tissue specimen number for blinded analysis.

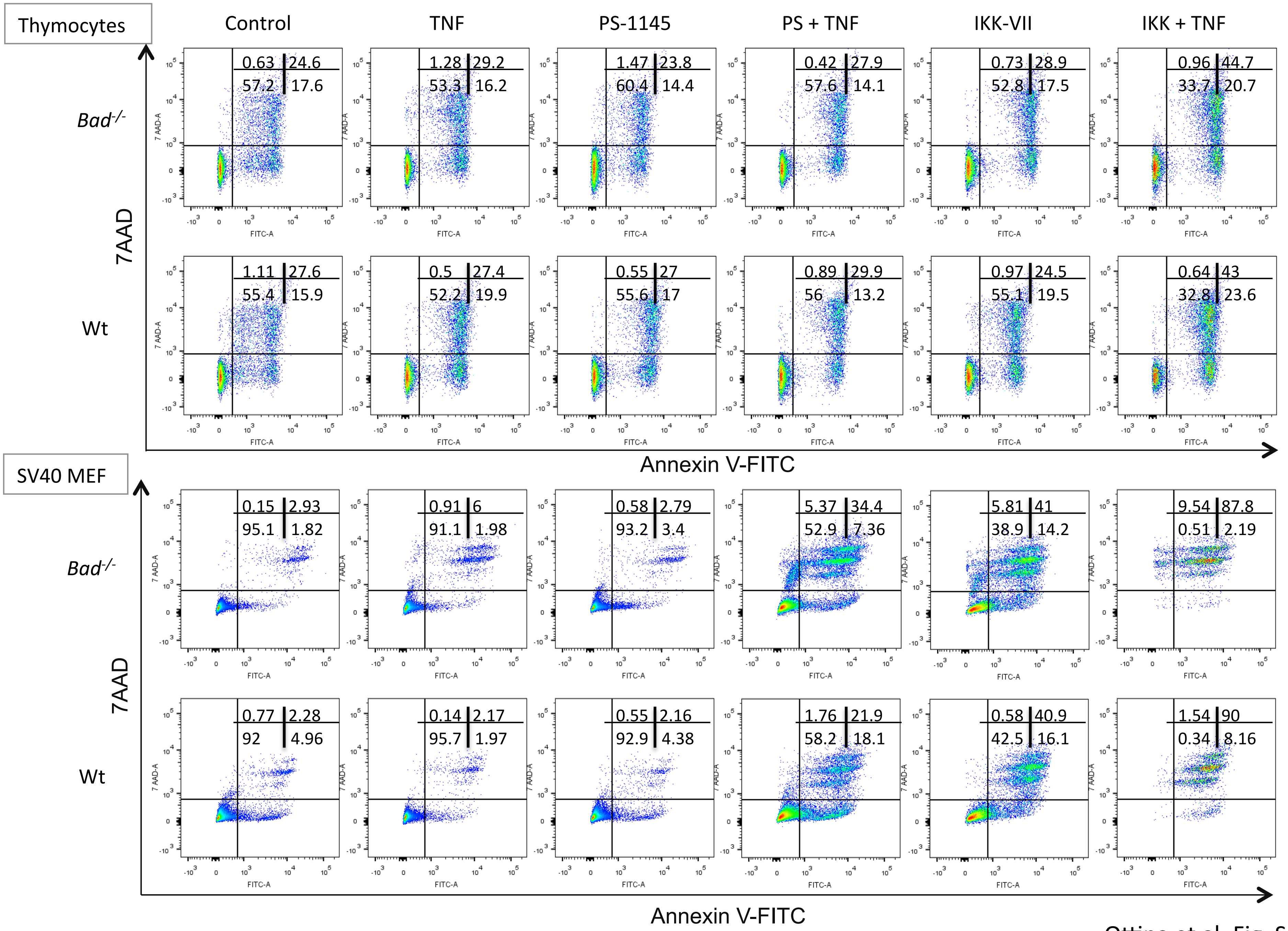

Ottina et al_Fig. S1

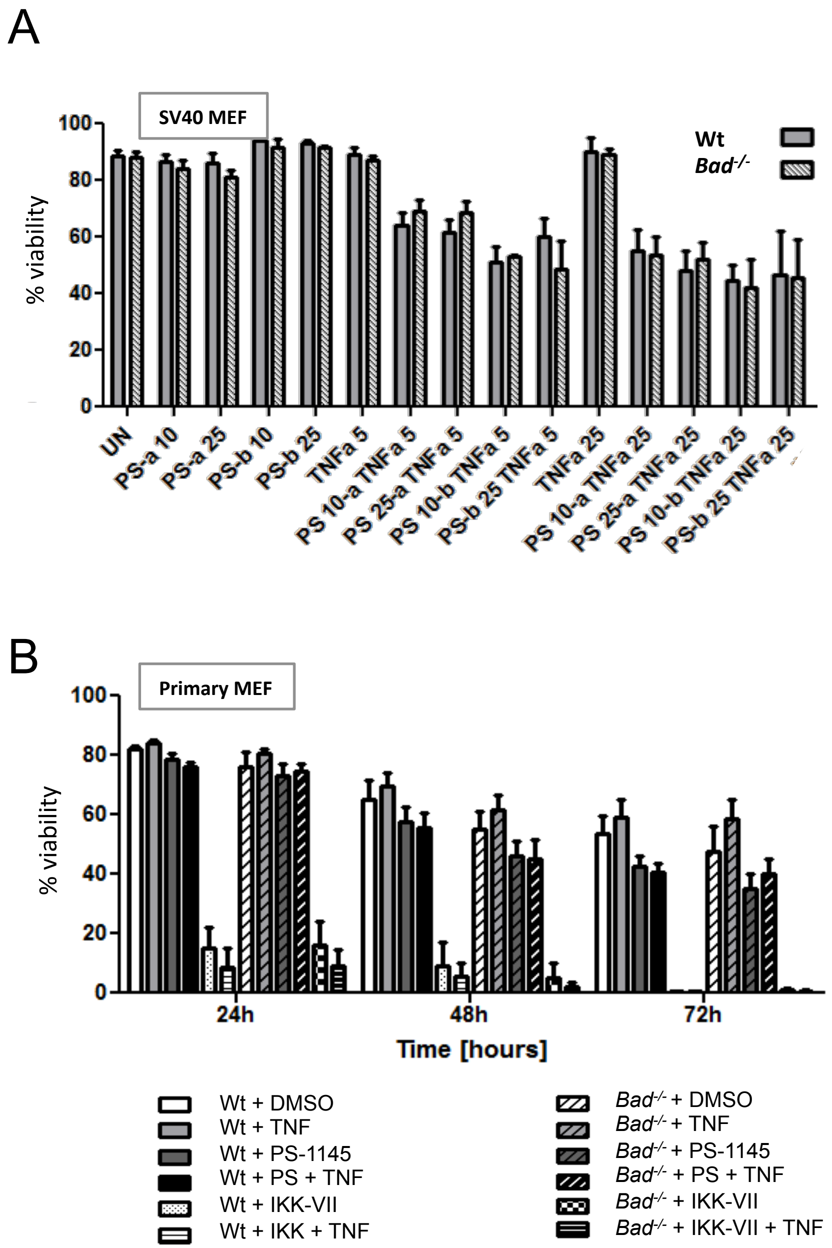

Ottina et al_Fig. S2

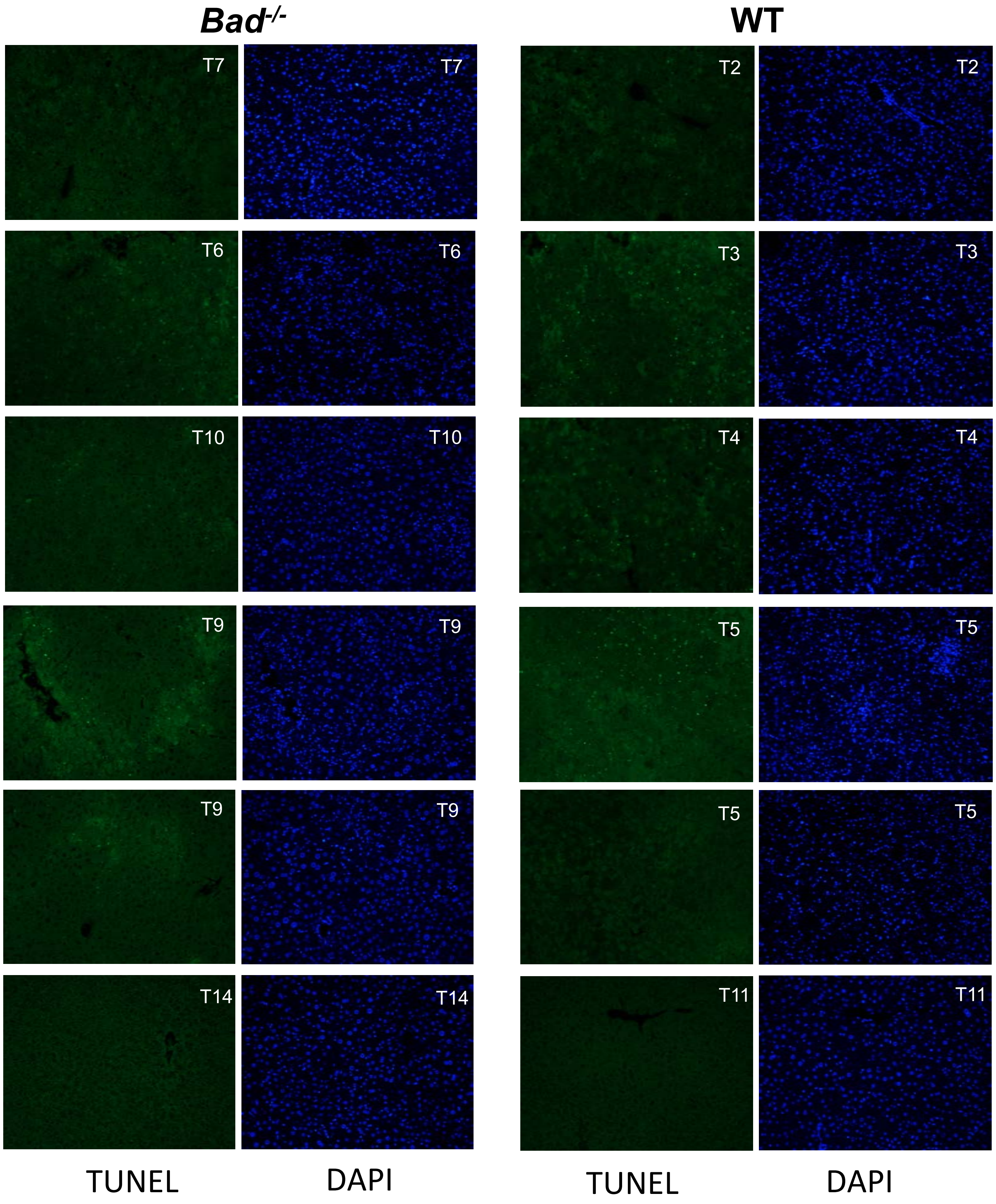

Bad-/-
WT
T7
T7
T6
T6
T10
T10
T9
T9
T9
T9
T14
T14
T2
T2
T3
T3
T4
T4
T5
T5
T5
T5
T11
T11
TUNEL
DAPI
TUNEL
DAPI
